# Dietary pea fiber increases diversity of colonic methanogens of pigs with a shift from *Methanobrevibacter* to *Methanomassiliicoccus*-like genus and change in numbers of three hydrogenotrophs

**DOI:** 10.1186/s12866-016-0919-9

**Published:** 2017-01-17

**Authors:** Yuheng Luo, Hong Chen, Bing Yu, Jun He, Ping Zheng, Xiangbing Mao, Gang Tian, Jie Yu, Zhiqing Huang, Junqiu Luo, Daiwen Chen

**Affiliations:** Key Laboratory for Animal Disease-Resistance Nutrition of China, Ministry of Education, Animal Nutrition Institute, Sichuan Agricultural University, Chengdu, China

**Keywords:** Pea fiber, Methanogen, Diversity, Colon, Pig

## Abstract

**Background:**

Pea fiber (PF) is a potential fibrous supplement in swine production. The influence of dietary PF on microbial community in the colon of pigs remains largely unexplored. Methanogens in the hindgut of monogastric animals play important roles in degradation of dietary fibers and efficient removal of microbial metabolic end product H_2_. Understanding the impact of dietary PF on the structure of colonic methanogens may help understand the mechanisms of microbe-mediated physiological functions of PF. This study investigated the influence of PF on the diversity and quantity and/or activity of colonic methanongens of piglets and finishing pigs. Four archaeal 16S rRNA clone libraries were constructed for piglets and finishers fed with control (Piglet-C and Finisher-C) or PF diet (Piglet-P and Finisher-P).

**Results:**

There were 195, 190, 194 and 196 clones obtained from the library Piglet-C, Piglet-P, Finisher-C and Finisher-P, respectively, with corresponding 12, 11, 11 and 16 OTUs (operational taxonomic units). Significant differences of Shannon Index among the four libraries were found (*P* < 0.05). Libshuff analysis showed that the archaeal community structure among the four libraries were significantly different (*P* < 0.0001). The predominant methanogens shifted from *Methanobrevibacter* to *Methanobrevibacter* and *Methanomassiliicoccus*-like genus as a result of dietary PF. Supplementation of PF significantly increased the copy numbers of *mcrA* and *dsrA* genes (*P* < 0.05).

**Conclusions:**

Alteration of methanogenic community structure may lead to functional transition from utilization of H_2_/CO_2_ to employment of both H_2_/CO_2_ and methanol/CO_2_. Quantification of three functional genes (*mcrA*, *dsrA* and *fhs*) of methanogens, sulfate-reducing bacteria (SRB) and acetogens revealed that dietary PF also increased the activity of methanogens and SRB,probably associated with increased proportion of *Methanomassiliicoccus luminyensis*-species. Further study is required to examine the interaction between specific methanogens and SRB during fermentation of dietary PF.

**Electronic supplementary material:**

The online version of this article (doi:10.1186/s12866-016-0919-9) contains supplementary material, which is available to authorized users.

## Background

Dietary fibers (DF) have been demonstrated to reduce incidence of intestinal diseases such as inflammatory bowel disease, colon cancer and diarrhea [1, 2]. DF that escape digestion in the small intestine pass largely intact into the colon where they function to increase viscosity and bulking of the fecal matter [3] and can be eventually degraded by colonic microbiota [4] in monogastric animals. With huge populations, gut microbes play a key role in digestion and absorption of nutrients and promotion of the host immune system [5]. As one of the 3 domains of microorganisms in the gastrointestinal (GI) tract, methanogens mostly colonize the colon of monogastric animals and use hydrogen to reduce carbon dioxide, formate or acetate to methane [6], thus helping keep the efficiency of microbial fermentation in the hindgut. Changes of dietary components can have direct and rapid impact on microbial community of the gut [7] as an adaptative mechanism. Of the dietary components, DF is proven to have a major impact on the composition of intestinal microbiota [3]. Although methanogens have been recognized as an important group of microorganisms in microbial fermentation, few studies were carried out to investigate the interaction of DF and methanogens in the GI tract of monogastric animals.

Recently, researchers from Thailand investigated the quantity of methanogens in the feces of children from two different geographic regions using quantitative PCR [8]. The children from northeastern Thailand had significantly higher consumption frequency of meat (chicken and beef), a wide variety of carbohydrate sources including noodle, fermented rice, sweet potato, vegetables and fruits. In central Thailand, there was significant preference for rice, breakfast cereal and cow milk. Although there was a large difference of food structures between children from the two regions, no significant alteration on the quantity of fecal methanogens was observed. Another study on humans [9] showed that the cellulose-degrading bacterial community differed in methane- and non-methane-excreting individuals, and the structure of the cellulolytic community varied with the presence of methanogens in the gut. Volunteers who ingested type III resistant starch (RS) and reduced carbohydrate weight loss (WL) diet harbored higher proportion of colonic methanogens than those who ingested maintained and non-starch polysaccharides (NSPs, 27.7 g/d) diets [10]. In healthy participants with detectable archaea (>106 copies per gram of feces), principal component analysis (PCOA) identified a distinct archaeal factor with positive loadings of age, breath methane, TDF, TDF/1000 kcal, and number of log archaea 16S rRNA gene copies [11]. In addition, some enteral condition such as *pH* can also influence the quantity and activity of methanogens. It is also reported that a high FODMAPs (Fermentable Oligo-, Di- and Mono-saccharides And Polyols) load led to greater production of short chain fatty acids and subsequent acidification of the lumen, which might then inhibit the activity of methanogens [12]. Some early studies suggest that differences in diet do not affect methane production, because it is largely dependent on substrates of endogenous origin [13, 14]. Other studies show that exogenous substrates like lactulose in various doses [15, 16] and dietary pentoses [17] can significantly increase methane production.

These limited studies on humans mostly focused on the interaction between dietary components/environment and the quantity/activity (methane production) of methanogens. Whether there are similar results in other monogastric animals, such as pigs, remains unknown. Moreover, there is a dispute on whether or how the dietary factors impact on gut methanogens.

Pea fiber (PF) has been shown to improve human health by regulating glucose response, lipid metabolism, and intestinal frequency [18]. Our previous study demonstrated that supplementation of a soluble dietary fiber, yeast derived β-glucan, significantly increased the diversity of methanogens in the swine colonic digesta in vitro and showed a beneficial effect on the growth of methanogens, which might improve microbial fermentation efficiency in the hindgut of pigs [19]. As PF is one of the widely utilized dietary fibers, we hypothesize that ingestion of PF probably increases the diversity, quantity and/or activity of colonic methanogens both in piglets and finishers. Therefore, we investigated the influence of PF on the diversity and community of colonic methanogens in piglets and finishing pigs (finishers in brief) using 16S rRNA gene clone library analysis. Because methanogens, sulfate-reducing bacteria (SRB) and acetogens are regarded as main potential competitors of H_2_, abundance of their functional genes, methyl-coenzyme M reductase (*mcrA*), dissimilatory sulfite reductase (*dsrA*) and formyltetrahydrofolate synthetase (*fhs*) was also examined to investigate the activity of the three H_2_ utilizing microbes.

## Methods

### Animal experiment and collection of samples

A total of 48 weaned pigs (Duroc × Landrace × Yorkshire, weaned at day 28) with an average initial body weight of 7.2 ± 0.5 kg were randomly allocated to 2 groups, PF-supplemented and control (without PF) groups as described in our previous study [18]. Pigs in the PF group were fed with diets containing 10% (30 d post-weaning), 20% (30–90 d post-weaning), or 30% (30–90 d post-weaning) of PF. The composition of diets and husbandry management has been described before [18].

At the end of the first (30 d post-weaning) and the third (160 d post-weaning) experimental period, twelve pigs in each group were sacrificed and the mid-colon tissues were removed immediately. Approximately 10 g digesta (divided into two sterilized 5-ml centrifuge tubes) from the mid-colon of each pig were immediately collected and stored at -80 °C for analysis of archaeal 16S rRNA clone library. Colonic digesta samples were classified according to their sources: Piglet-C and Finisher-C for samples from piglets and finishers in the control group, and Piglet-P and Finisher-P for samples from pigs in PF group.

### DNA extraction, clone library construction and phylogenetic analysis

Nucleic acids for each sample were extracted from 0.5 g of wet colonic digesta using the bead-beating method described before [20]. The DNA samples were purified with a PCR Clean-Up system (Promega, Madison, USA) and stored at -20 °C for later analysis. PCR amplification for Archaeal 16S rRNA genes and construction of the clone libraries were carried out according to described methods [21]. A total of 200 transformed clones with correct sized inserts were selected and sequenced (Invitrogen, Shanghai, China).

For each clone library, chimeras of the sequences and assignment of 16S rRNA gene sequences into operational taxonomic units (OTUs) were analyzed with the software MOTHUR (ver 1.23.1) [22]. Coverage of each clone library was calculated according to the equation C = 1 - (n/N) [23]. GenBank’s Basic Local Alignment Search Tool (BLAST) [24] and the phylogenetic software PHYLIP (ver 3.69) [24] was used to identify the nearest validly described neighbor of each sequence and construct the neighbor-joining tree, respectively.

### Real-time PCR for abundance of methanogen, sulfate-reducing bacteria, acetogens, *Methanobrevibacter* genus, *M. smithii* and *M. boviskoreani*

Abundance of methanogen, SRB, acetogens, *Methanobrevibacter*, *M. smithii* and *M. boviskoreani* were quantified by real-time PCR on a BioRad CFX-96 real time system (BioRad, USA) using SYBR Green as the fluorescent dye. The copies of *mcrA*, *dsrA* and *fhs* genes (for methanogen, SRB and acetogens, respectively) were determined with primer sets qmcrA-F/qmcrA-R [25], Drs1 + -F/Dsr-R [26] and fhs1/FTHFS-r [27], respectively. Abundance of *Methanobrevibacter* [28], *M. smithii* [29] and *M. boviskoreani* [30] were determined with primer sets described previously. A total of 25 μl reaction mixture contained 12.5 μl of IQ SYBR Green Supermix (Bio-Rad), 0.2 μM of primer sets and 5 μl of DNA template. The copies of each gene in each sample were determined in triplicate, and the mean values were calculated. Standard curve of each gene was generated by using the serial dilutions of purified PCR amplicon.

### Statistical analysis

Copy numbers of *mcrA*, *dsrA* and *fhs* genes for group Piglet-C, Piglet-P, Finisher-C and Finisher-P were analyzed with One-way Analysis of Variance (ANOVA) program using the statistical software SPSS 16.0. The numbers of *Methanobrevibacter*, *M. smithii* and *M. boviskoreani* between Piglet-C and Piglet-P, or Finisher-C and Finisher-P were compared with Paired Samples *T*-test. Results were expressed as the mean ± SD. Differences were considered as significant when *P* value is less than 0.05.

## Results

### The diversity of methanogens in the four clone libraries

**Fig. 1:**
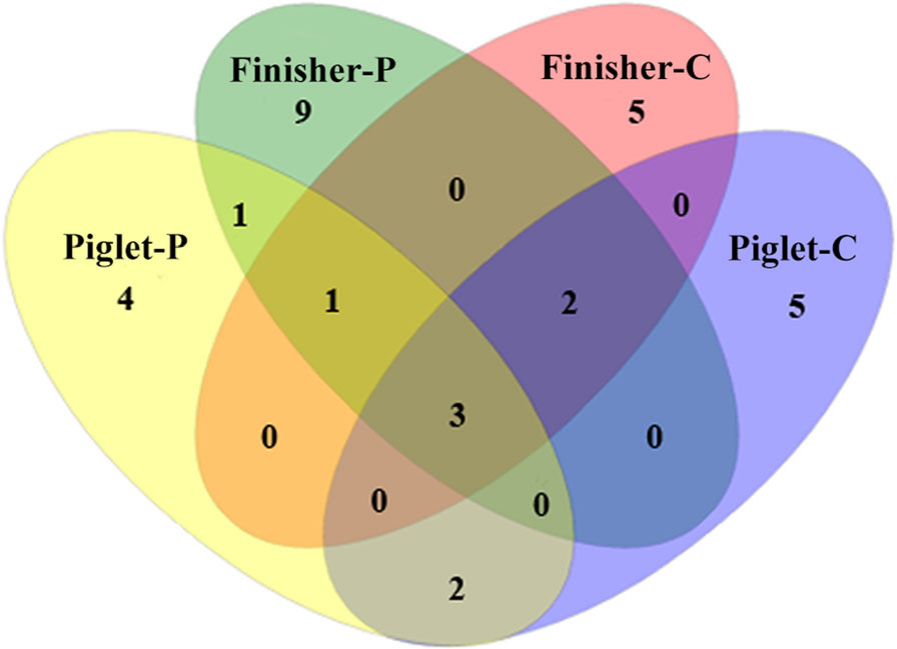
Venn diagram of the four clone libraries. Five OTUs were shared between group Piglet-C and Piglet-P, six between Finisher-C and Finisher-P, and five OTUs between Piglet-C and Finisher-C, or Piglet-P and Finisher-P

A total of 775 qualified sequences were obtained from the 800 cloned archaeal 16S rRNA amplicons and included 195, 190, 194 and 196 clones from libraries Piglet-C, Piglet-P, Finisher-C and Finisher-P, respectively. Sequence examination of these clones revealed a total of 32 OTUs (Table 1). The 195 sequences from group Piglet-C were classified into 44 different phylotypes (Additional file 1: Table S1) and 12 OTUs based on a 98% sequence identity criterion. The 190 sequences from Piglet-P library were assigned to 40 phylotypes and 11 OTUs (Additional file 1: Table S1). The 194 sequences from Finisher-C library were identified into 41 phylotypes and 11 OTUs. Those of the Finisher-P library were assigned to 43 phylotypes and 16 OTUs (Additional file 1: Table S1). There were five OTUs shared between libraries Piglet-C and Piglet-P, Piglet-C and Finisher-C, and Piglet-P and Finisher-P, respectively. Six OTUs were shared between libraries Finisher-C and Finisher-P (Fig. 1, Table 1).

**Table 1:** Operational taxonomic units (OTUs) of archaeal 16S rRNA gene sequences from colonic digesta of piglets and finishers

OTU#	#Phylotype	#Sequence	Nearest Valid Taxon^a^	% Sequence Identity
1	1	28	*Methanobrevibacter smithii*	99.1
2	1	4	*Methanobrevibacter millerae*	98.7
3	1	3	*Methanobrevibacter millerae*	98.1
4	2	7	*Methanobrevibacter millerae*	98.6
5	1	8	*Methanobrevibacter boviskoreani*	99.4
6	2	5	*Methanobrevibacter millerae*	98.2
7	1	27	*Methanomassiliicoccus luminyensis*	86.6
8	1	1	*Methanobrevibacter ruminantium*	96.9
9	1	2	*Methanobrevibacter boviskoreani*	99.7
10	2	11	*Methanobrevibacter millerae*	98.3
11	1	16	*Methanomassiliicoccus luminyensis*	84.6
12	1	4	*Methanobrevibacter ruminantium*	96.4
13	1	3	*Methanobrevibacter ruminantium*	97.7
14	2	18	*Methanobrevibacter millerae*/*Methanobrevibacter smithii*	96.7
15	1	15	*Methanomassiliicoccus luminyensis*	88.0
16	1	1	*Methanobrevibacter millerae*	95.5
17	4	33	*Methanomassiliicoccus luminyensis*	88.2
18	1	2	*Methanomassiliicoccus luminyensis*	87.3
19	1	8	*Methanomassiliicoccus luminyensis*	84.3
20	2	5	*Methanomassiliicoccus luminyensis*	87.4
21	1	2	*Methanobrevibacter millerae*	96.1
22	1	5	*Methanobrevibacter gottschalkii*	97.9
23	1	4	*Methanobrevibacter gottschalkii*	97.5
24	1	1	*Methanobrevibacter gottschalkii*	97.6
25	5	33	*Methanobrevibacter millerae*	98.4
26	9	23	*Methanobrevibacter millerae*	97.8
27	4	14	*Methanobrevibacter gottschalkii*	98.3
28	10	59	*Methanobrevibacter ruminantium*	98.9
29	1	19	*Methanomassiliicoccus luminyensis*	86.7
30	100	353	*Methanobrevibacter millerae*/*Methanobrevibacter gottschalkii*	98.6
31	6	52	*Methanobrevibacter olleyae*/*Methanobrevibacter millerae*/*Methanobrevibacter smithii*	96.1
32	1	9	*Methanobrevibacter gottschalkii*	98.3
Totals	168	775		

^a^Nearest valid taxon with the same name means the same strain

The coverage of the four libraries was from 98.95% to 99.49% (Table 2). The values of observed and estimated OTUs for each library were close to each other or with a variance less than 0.5 (Table 2). There were significant differences of the Shannon Index among the four libraries (*P* < 0.05). Libshuff analysis showed significant differences of methanogen community structure among the four libraries (*P* < 0.0001) (Table 2).

**Table 2:** Coverage and Shannon Index calculated using MOTHUR1 for each methanogen 16S rRNA gene clone library

Clone library	OTUs observed	CHAO1 OTU estimate	% OTU coverage^2^	Shannon index ± 95% confidence limits	Libshuff analysis
Piglet-C	10	10	99.49	1.47 ± 0.15^a^	*P <* 0*.*0001
Piglet-P	11	11.5	98.95	1.53 ± 0.15^b^	*P <* 0*.*0001
Finisher-C	10	10	99.48	1.66 ± 0.13^c^	*P <* 0*.*0001
Finisher-P	15	15	99.49	2.08 ± 0.16^d^	*P <* 0*.*0001

^1^Schloss et al

^2^Good’s coverage (*C*) according to the equation *C* = 1 *−* (*n*/*N*), where *n* is the number of sequences represented by a single clone and *N* is the total

number of clones in the library

^a,b,c,d^ There is significant difference between these values

### The community structure of methanogens in the four clone libraries

**Fig. 2:**
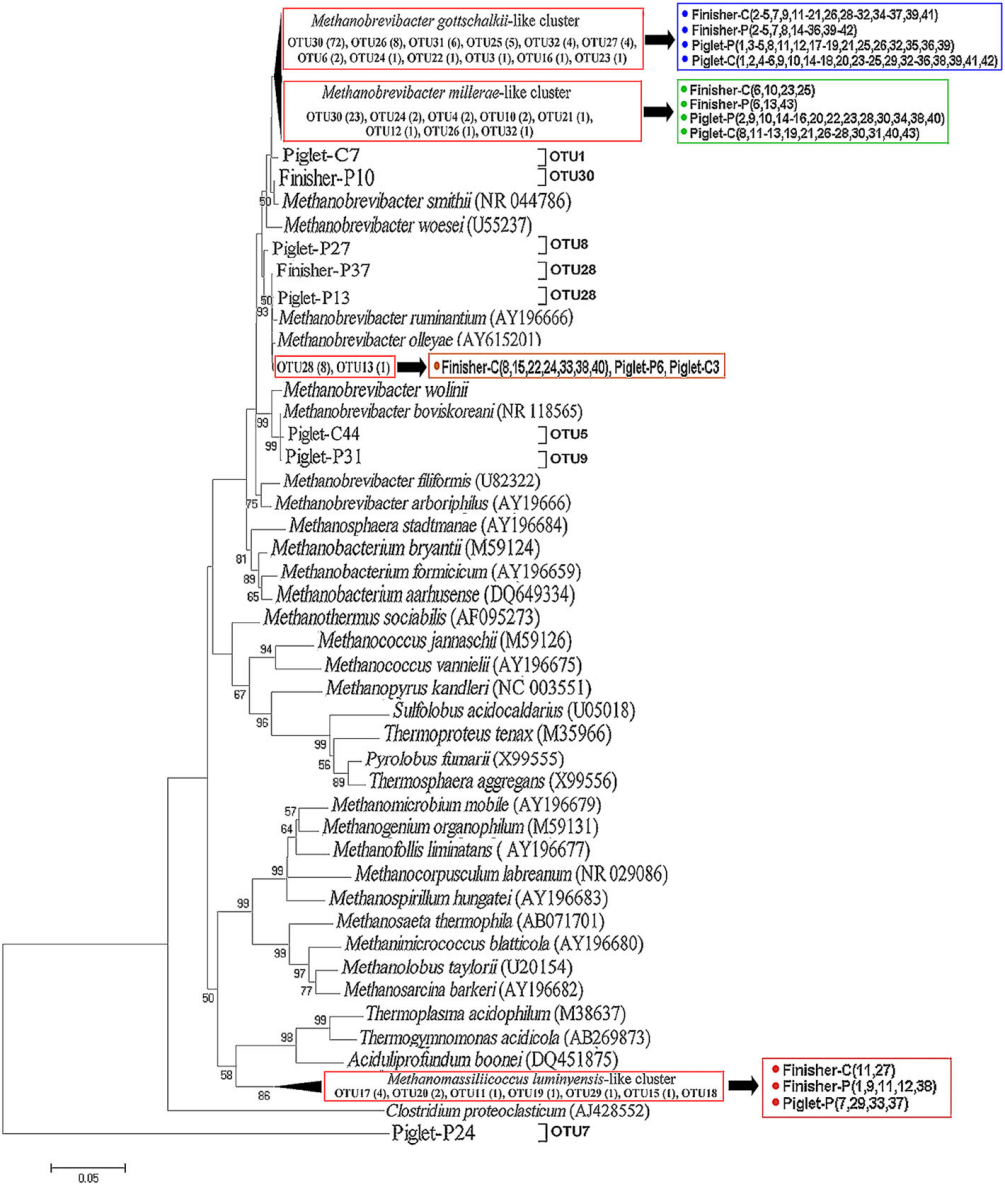
Phylogenetic relationship of archaeal 16S rRNA gene sequences retrieved from colonic samples of piglets and Finishers. Evolutionary distances were calculated using the Neighbor-Joining method. The tree was bootstrap resampled 1000 times

**Fig. 3:**
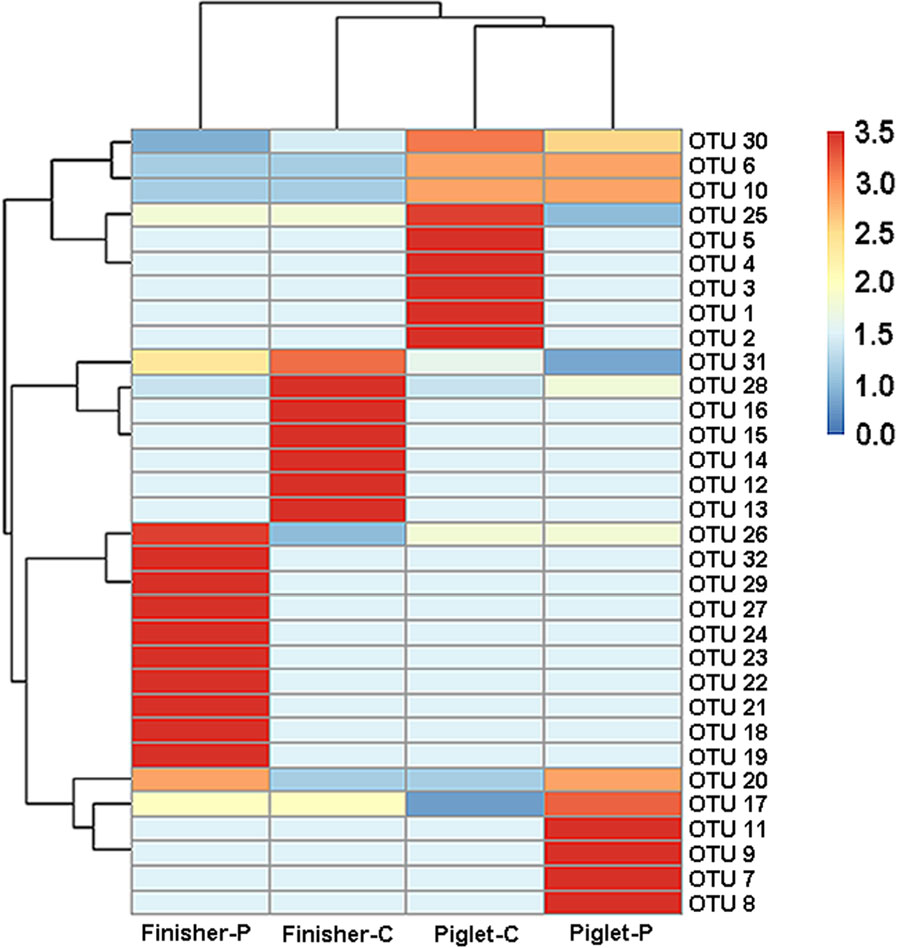
Heatmap distribution of OTUs of methanogens for all colonic samples. OTUs are arranged in rows and clustered on the vertical axis (y-axis). Groups are arranged vertically and are on the horizontal axis (x-axis). Clustering was done for each using Phylotrac’s heatmap option with Pearson correlations and complete lineage algorithms. Different colors indicate relative abundance of the taxons

All sequences in the four libraries were identified to the phylum Euryarchaeota. Majority of the sequences (71%) in library Piglet-C were assigned to 8 different OTUs (OTU-2, OTU-3, OTU-4, OTU-6, OTU-10, OTU-25, OTU-26 and OTU-30) and closely related to species *Methanobrevibacter millerae* with sequence identities ranging from 97.2% to 98.7%. Twenty-eight sequences (14%) were assigned to OTU-1 and closely related to *M. smithii* (99.1%). OTU-25 with 18 sequences (9%) was closely related to *M. gottschalkii* with 98.4% identity, and OTU-5 with 8 sequences (4%), to *M. boviskoreani* with 99.4% identity. Only OTU-28 (2 sequences) was closely related to *M. ruminantium* (98.5%) (Fig. 2, Fig. 3, Additional file 1: Table S1).

About half of the sequences (48%) in library Piglet-P were assigned to 4 OTUs (OTU-6, OTU-10, OTU-26 and OTU-30) and closely related to species *M. millerae* with similarities from 97.1% to 98.6%. Sixty-nine sequences (36%) were assigned to 4 OTUs (OTU-7, OTU-11, OTU-17 and OTU-20) with lower similarities (from 84.6% to 88.1%) and related to *Methanomassiliicoccus luminyensis*. Nineteen sequences (10%) were assigned to 2 OTUs (OTU-8 and OTU-28) and related to *M. ruminantium* with similarities from 96.9% to 98.9%. Eight sequences belonging to OTU-30 showed 97.9% to 98.3% relatedness to *M. gottschalkii*. Only two sequences assigned to OTU-9 were closely related to *M. boviskoreani* (99.7%)(Fig. 2, Fig. 3, Additional file 1: Table S1).

In library Finisher-C, nearly half of the sequences (47%) were assigned to 5 OTUs (OTU-14, OTU-16, OTU-25, OTU-26 and OTU-30) and related to *M. millerae* with similarities ranged from 95.5% to 98.4%. One fifth of the sequences (20%) assigned to 3 OTUs (OTU-12, OTU-13 and OTU-28) were affiliated to species *M. ruminantium* with similarities from 96.4% to 98.8%. Thirty-seven sequences (19%) belonging to OTU-14 and OTU-31 showed relatedness to *M. smithii* with lower identities (96.0% to 96.7%). Eighteen sequences (9%) were assigned to OTU-15 and OTU-17 and related to *Methanomassiliicoccus luminyensis* with lower similarities from 88.0% to 88.2%. In addition, nine sequences were assigned to OTU-31 and related to *Methanobrevibacter olleyae* with 96.1% identity (Fig. 2, Fig. 3, Additional file 1: Table S1).

There were 29% sequences in library Finisher-P assigned to 5 OTUs (OTU-21, OTU-25, OTU-26, OTU-30 and OTU-31) and related to *M. millerae* with identities ranging from 96.1% to 98.4%. Forty sequences (20%) were assigned also to 5 OTUs (OTU-17, OTU-18, OTU-19, OTU-20 and OTU-29) and related to *Methanomassiliicoccus luminyensis* with low identities from 84.3% to 88.2%. Thirty-four sequences (17%) were assigned to 6 OTUs (OTU-22, OTU-23, OTU-24, OTU-27, OTU-30 and OTU-32) and closely (from 97.5% to 98.3%) related to *M. gottschalkii*. Additionally, thirty-eight, twenty-one and seven sequences were assigned and corresponded to OTU-30, OTU-31 and OTU-28, and related to *M. smithii* (98.6%), *M. olleyae* (98.7%) and *M. ruminantium* (96.1%), respectively (Fig. 2, Fig. 3, Additional file 1: Table S1).

### Abundance of total methanogens (*mcrA*), SRB (*dsrA*), acetogens (*fhs*) and specific methanogenic species in the colonic samples

**Fig. 4:**
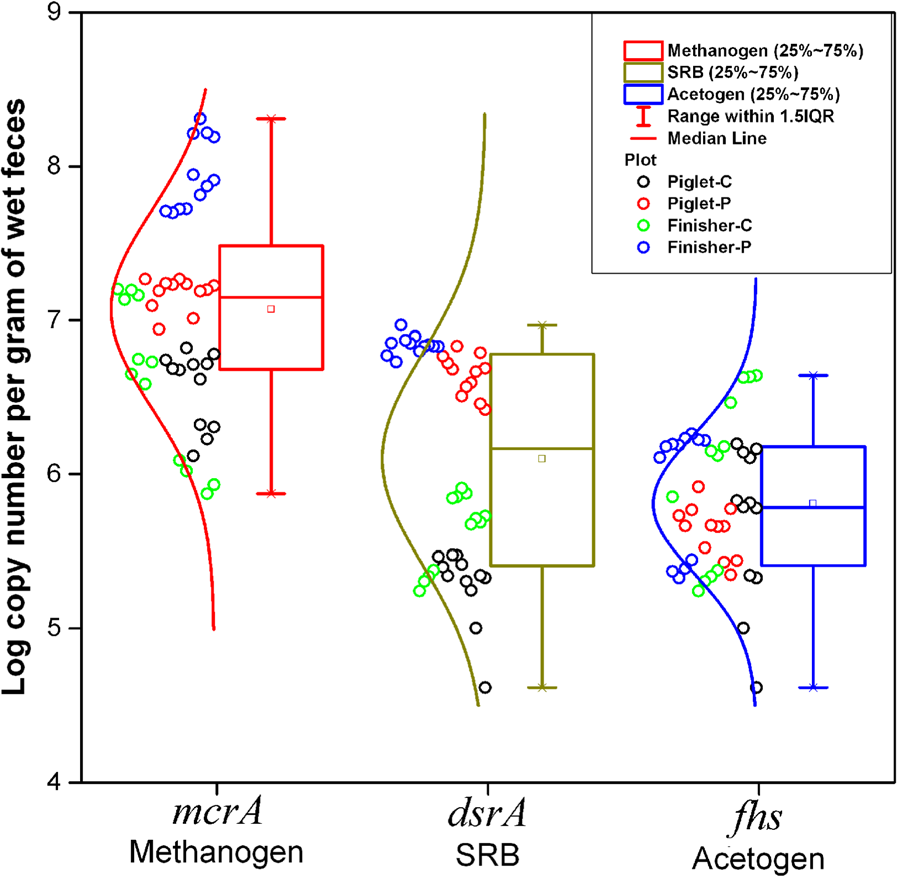
The interquartile range (IQR) diagram based on the qPCR data for the *mcrA*, *dsrA* and *fhs* genes

**Fig. 5:**
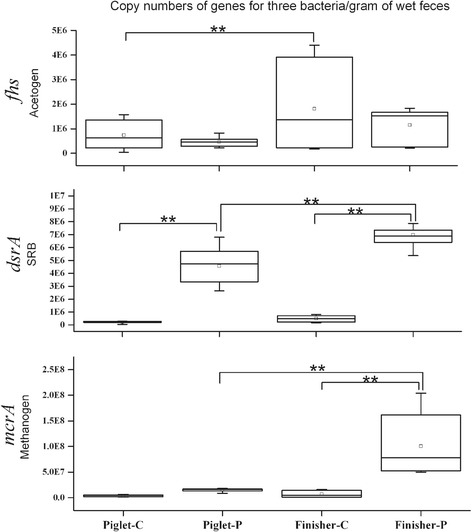
The *box* plot based on the qPCR data for the *mcrA*, *dsrA* and *fhs* genes. Significant differences (*P* < 0.01) are shown on the plot as “**”

The inter quartile range (IQR) analysis showed that the qPCR data of *mcrA*, *dsrA* and *fhs* genes for samples from each group were relatively concentrated (Fig. 4). The copy number of the *mcrA* gene in samples from group Finisher-C and Piglet-P was significantly lower than Finisher-P (*P* < 0.01). Thst of the *dsrA* gene in samples from Piglet-C was significantly lower than Piglet-P (*P* < 0.01). Both Finisher-C and Piglet-P samples had lower copies of the *dsrA* gene than Finisher-P (*P* < 0.01). The copy number of the *fhs* gene in samples from group Piglet-C was significantly lower than Finisher-C (*P* < 0.01) (Fig. 5).

Compared to group Piglet-C, significant increase was seen in abundance of the genus *Methanobrevibacter* (*P* < 0.01) and *M. boviskoreani* (*P* < 0.05) in samples from group Piglet-P, while marked reduction was observed in the number of *M. smithii* and the ratio of *Methanobrevibacter* to total methanogens (*P* < 0.01). In addition, the number of genus *Methanobrevibacter* and *M. smithii* was significantly (*P* < 0.01) higher in samples from Finisher-P than those from Finisher-C with significantly decreased ratio of *Methanobrevibacter* to total methanogens (*P* < 0.05) (Table 3).

**Table 3:** The copy numbers of *Methanobrevibacter*, *M. smithii* and *M. boviskoreani* in samples from pigs of the four groups

Methanogen group	Piglet-C	Piglet-P	*P*-Value	Finisher-C	Finisher-P	*P*-Value
*Methanobrevibacter*	4.00 × 10^6^ ± 2.43 × 10^6^	9.43 × 10^6^ ± 1.81 × 10^6^	0.00	6.90 × 10^6^ ± 5.55 × 10^6^	1.00 × 10^8^ ± 6.17 × 10^6^	0.00
*M. smithii*	4.82 × 10^5^ ± 2.51 × 10^5^	2.58 × 10^4^ ± 4.72 × 10^4^	0.00	1.29 × 10^6^ ± 1.46 × 10^6^	1.88 × 10^7^ ± 1.09 × 10^7^	0.00
*M. boviskoreani*	1.47 × 10^5^ ± 2.51 × 10^5^	2.39 × 10^5^ ± 1.60 × 10^5^	0.03	1.93 × 10^4^ ± 5.79 × 10^4^	4.35 × 10^4^ ± 1.44 × 10^5^	0.61
*Methanobrevibacter*%	0.97 ± 0.03	0.61 ± 0.07	0.00	0.87 ± 0.08	0.78 ± 0.05	0.02

The real-time PCR data are shown as Mean ± Standard Deviation (SD)

## Discussion

The influence of DF to the diversity and activity of colonic methanogens is poorly understood. To the best of our knowledge, this study is the first to report that dietary PF had extensive influence on the community structure of methanogens in the colon of pigs. The rarefaction curve (Additional file 1: Figure S1) and high coverage of the clone libraries (≥98.95%) indicated that the libraries were well sampled and the results based on sequencing analysis were representative. We clearly show that dietary PF increased the diversity of colonic methanogens in pigs. Similar result was observed in our previous in vitro study that yeast β-glucan significantly increased the archaeal diversity in fermented colonic digesta of pigs [19].

In the colon of piglets fed with basal diet, all archaeal sequences belonged to genus *Methanobrevibacter. M. millerae* and *M. millerae*-like species were the predominant methanogens (70.77% of the total sequences), followed by *M. smithii*, *M. gottschalkii*, *M. boviskoreani*, *M. ruminantium* and *M. olleyae* (Table 4). However, in the colonic digesta of piglets fed with PF diet, sequences belonging to *M. millerae* and *M. gottschalkii* were decreased with increased ratio of *M. ruminantium* associated sequences. *Methanomassiliicoccus luminyensis*, which was not found in piglets receiving control diet, was the second predominant methanogen in the colon of piglets fed with PF diet. *M. smithii* and *M. olleyae* were not detected in PF-fed piglets (Table 4, Additional file 1: Figure S2). Interestingly, the change of methanogen species between control and PF-fed piglets was different to some degree from the finishers. *M. millerae* was the predominant species in the colon of finishers from both control and PF groups. Although *Methanomassiliicoccus luminyensis* was found in finishers from both groups, its ratio in the colon of PF pigs was 11.13% higher than the control. *M. gottschalkii* was found only in PF-fed pigs, and *M. boviskoreani* was not detected from both groups (Table 4). Unlike piglets, both *M. smithii* and *M. olleyae* were increased in PF-fed finishers (Table 4, Additional file 1: Figure S2). A few studies reported that *Methanobrevibacter* was the main methaongen genus in the hindgut of humans and most monogastric animals [5, 31–33], in which *Methanobrevibacter* and *M. smithii* were predominant [31, 34]. Our study also revealed predominance of *Methanobrevibacter*, but not *M. smithii*, in the colon of pigs.

**Table 4:**
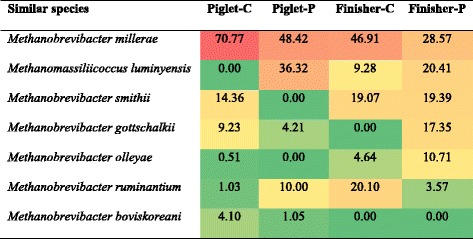
The proportion of most similar species in the four clone libraries (%)

The background color of each cell indicates relative abundance of each phylum with red and green indicating highest and lowest values

Methanogens belonging to the genus *Methanobrevibacter*, such as *M. millerae*, *M. olleyae* and *M. boviskoreani*, can utilize the end products of bacterial fermentation in the hindgut to produce methane from H_2_/CO_2_ or from formate plus CO_2_ [35]. While *Methanomassiliicoccus luminyensis*, a newly isolated methanogen from human feces, produces methane by reducing methanol with hydrogen as the electron donor, it can not produce methane when hydrogen or methanol is the sole energy source [36]. Predominance of *Methanobrevibacter* in pigs from control group suggests that in the hindgut of these pigs, the methanogen species probably has preference to using H_2_ or formate and CO_2_ as substrates. The fact that dietary PF decreased the ratio of *Methanobrevibacter* to total methanogens (shown by qPCR) indicaties a relative increase of other methanogen genera. Predominance of *Methanomassiliicoccus luminyensis*-like species in the PF-fed pigs suggests that the available substrates for methanogens in the colon of these pigs might become more diverse and shift from H_2_/CO_2_ and formate/CO_2_ to only menthanol/CO_2_. All these results indicate that PF supplementation in the diet can change the methanogen community structure in the colon of pig and render the methanogens more adaptable to different substrates for more efficient microbial fermentation, an indication on the response of methanogen community to dietary fiber in the hindgut of monogastric animal. The underlying mechanisms require further studies.

This study also considered the time effect of dietary PF to colonic methanogen community structure. In the colon of piglets at 30-d or finishers at 160-d old fed with control diet, *Methanobrevibacter* had absolute predominance. In PF-fed pigs, *Methanobrevibacter* was partially replaced by *Methanomassiliicoccus*-like genus at both age groups. Nevertheless, the proportion of *Methanomassiliicoccus luminyensis*-like species was higher in piglets (36.32%) than in finishers (20.41%) fed with PF-containing diet (Table 4). These findings suggest that dietary PF probably favors the presence of *Methanomassiliicoccus*-like genus in piglets and finishers. This change in the methanogen community may be involved in the transition of their functions. Only one study found that the archaeal population shifted during weaning and *M. boviskoreani* replaced *M. smithii*, as shown by denaturing gradient gel electrophoresis (DGGE) of *mcrA* gene [30]. However, we found a shift with decrease of *M. boviskoreani* and increase of *M. smithii* from piglets to finishers when fed on basal diet. In the PF group, *M. boviskoreani* was even not detected in finishers, while *M. smithii* increased from null to 19.39%. We had no explanation for this shift.

Archaeal sequences that are closely related to *M. smithii*, *M. gottschalkii*, *M. millerae* or *M. thaurei* are referred to as the *smithii*-*gottschalkii*-*millerae*-*thaurei* (SGMT) clade and those related to *M. ruminantium* and *M. olleyae*, as the *ruminantium*-*olleyae* (RO) clade. Distribution of SGMT and RO may vary with animal species and diets [37]. There were limited studies that focused mainly on the distribution of SGMT and RO in different ruminants [37, 38]. This study shows that SGMT is the predominant clade in the colon of all pigs. Compared with the control animals, the ratio of SGMT was markedly decreased (-41.73%) with increased RO clade in the colon of PF-fed piglets, while there was no apparent change of SGMT (-0.67%) and a decrease of RO in the colon of PF-fed finishers (Table 5, Additional file 1: Figure S3). During growth of the pigs in the control group, the SGMT clade was decrease relative to the change of RO. Supplementation of PF increased both SGMT and RO clades in the colon of finishers. Further study is needed to find out whether there is functional relationship between methanogens and their distribution of SGMT and RO clades.

**Table 5:**
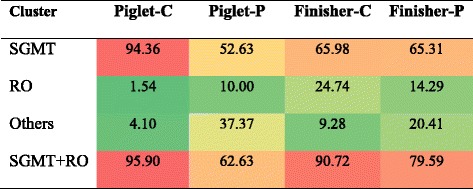
The proportion of most closely related methanogenic clades in the four clone libraries (%)

The background color of each cell indicates relative abundance of each phylum with red and green indicating highest and lowest values

**Fig. 6:**
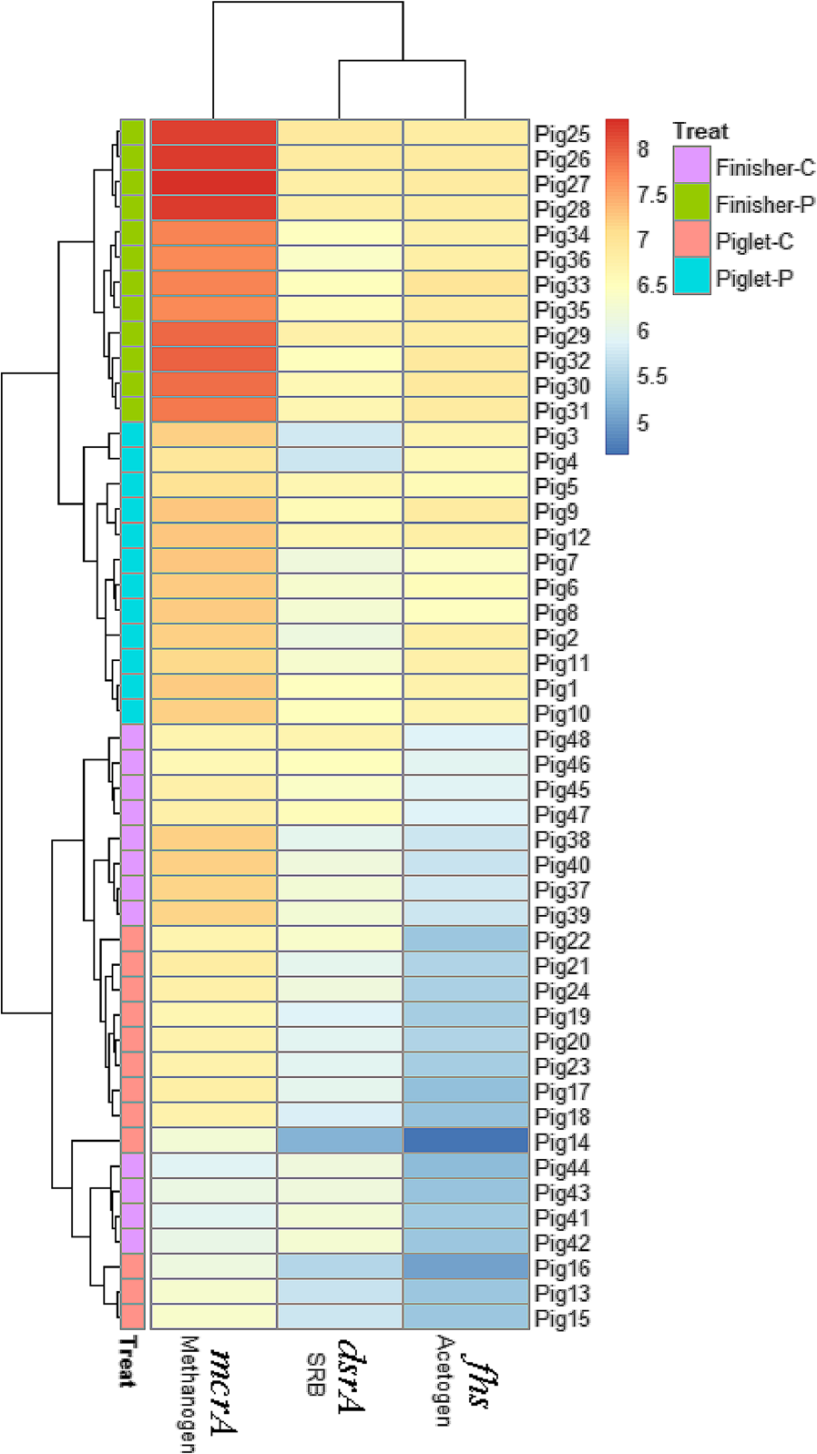
Heatmap distribution of copy numbers of *mcrA*, *dsrA* and *fhs* genes for all colonic samples based on qPCR data. Samples (*mcrA*, *dsrA* and *fhs* genes) are arranged in rows and clustered on the vertical axis (y-axis). Gene copies are arranged vertically and are on the horizontal axis (x-axis). Clustering was done for each using Phylotrac’s heatmap option with Pearson correlations and complete lineage algorithms. Different colors indicate relative abundance of the taxons. Different colors showed on the left side of the diagram indicate the four groups (Piglet-C, Piglet-P, Finisher-C and Finisher-P)

Accumulation of the end metabolic products, such as H_2_, CO_2_ and methanol, produced by colonic bacteria of monogastric animals can influence the fermentation efficiency of gut microbes [39]. Methanogens, SRB and acetogens are recognized as three important hydrogenotrophic microbes to assure fermentation efficiency through utilization of the end products [40–42]. There is competition among these hydrogenotrophic microbes [43]. Three unifying group specific genes, *mcrA*, *dsrAB* and *fhs*, are regarded as important functional genes which encode key enzymes involved in H_2_ consumption, namely, methyl-coenzyme M reductase, dissimilatory sulfite reductase, and formyltetrahydrofolate synthetase for methanogens, SRB and acetogens, respectively [44]. Thus, the quantity of these functional genes can reflect the activity of three hydrogenotrophic microbes. This study shows that dietary PF increased the activity of SRB in piglets and enhanced (Additional file 1: Table S2, Fig. 6) the activity of both methanogens and SRB in finishers. *Methanomassiliicoccus luminyensis*-like species, the main methane producer using only methanol and H_2_, occupied a large proportion of methanogens in the colon of PF-fed pigs. Methanol is of particular interest as electron donor because it is readily available and cost effective [43, 45]. The fate of methanol in anaerobic reactors is determined by the outcome of direct competition between methanogens, SRB and homoacetogens [46]. SRB are the most efficient hydrogenotrophs when sulfate is used as the electron acceptor. Under pure culture conditions, the H_2_ threshold (lowest concentration of H_2_ that can be used) of SRB is significantly lower than the average threshold of acetogens and methanogens [47]. Therefore, our results further indicate that alteration of dietary components, such as PF supplementation, impacted the end products of colonic bacteria, which in turn leads to competition of methanogens and SRB. Surprisingly, the activity of acetogens increased from piglets to finishers in control group, indicating that the increased activities of methanogens and SRB are more important than acetogens for degradation of dietary PF.

## Conclusions

Dietary PF increased the diversity of colonic methanogen community structure of pigs with a shift from *Methanobrevibacter* to *Methanobrevibacter* and *Methanomassiliicoccus*-like genus. This alteration may probably lead to functional transition, that is, utilization from H_2_/CO_2_ to both H_2_/CO_2_ and methanol/CO_2_. Moreover, dietary PF also increased the activity of methanogens and SRB, probably associated with increased proportion of *Methanomassiliicoccus luminyensis*-species. Further study on the interaction between methanogenic and SRB species during fermentation of dietary PF is needed.

## Additional file

Additional file 1: Supporting Data. (DOCX 159 kb)

## Abbreviations

DF: Dietary fibers
dsrA: Dissimilatory sulfite reductase
fhs: Formyltetrahydrofolate synthetase
FODMAPs: Fermentable Oligo- Di- and Mono-saccharides And Polyols
mcrA: methyl-coenzyme M reductase
OTUs: Operational taxonomic units
PF: Pea fiber
SRB: Sulfate-reducing bacteria
