# Supplementary material for: Dietary pea fiber increases diversity of colonic methanogens of pigs with a shift from Methanobrevibacter to Methanomassiliicoccus-like genus and change in numbers of three hydrogenotrophs
Source: BMC Microbiol. 2017 Jan 17;17:17. doi: 10.1186/s12866-016-0919-9 (PMC5240297; doi:10.1186/s12866-016-0919-9)
Supplement: Additional file 1: — Supporting Data. (DOCX 159 kb) [file 12866_2016_919_MOESM1_ESM.docx]

**Journal name**: BMC Microbiology

**Manuscript Title**: Dietary pea fiber increases colonic methanogen diversity with a shift from *Methanobrevibacter* to *Methanomassiliicoccus*-like genus and change in numbers of three hydrogenotrophs in pigs

**The names of the authors**: Yuheng Luo, Hong Chen, Bing Yu, Jun He, Ping Zheng, Xiangbing Mao, Gang Tian, Jie Yu, Zhiqing Huang, Junqiu Luo, Daiwen Chen

**The affiliations and addresses of the authors**: Key Laboratory for Animal Disease-Resistance Nutrition of China, Ministry of Education, Animal Nutrition Institute, Sichuan Agricultural University, Chengdu, China

**The e-mail address, telephone and fax numbers of the corresponding author**: Professor Daiwen Chen, email [dwchen@sicau.edu.cn](mailto:dwchen@sicau.edu.cn), Tel & Fax 00 86 0835 2882088

**Table S1**. Operational taxonomic units (OTUs) and phylotypes of archaeal 16S rRNA gene sequences in the four clone libraries.

| **Clone library** | **Phylotype** | **OTU#** | **#Sequence** | **Nearest Valid Taxon*** | **% Sequence Identity** |
| --- | --- | --- | --- | --- | --- |
| Piglet-C | Piglet-C1 | OTU30 | 2 | *Methanobrevibacter millerae* | 98.3 |
|  | Piglet-C2 | OTU6 | 4 | *Methanobrevibacter millerae* | 98.2 |
|  | Piglet-C3 | OTU28 | 2 | *Methanobrevibacter ruminantium* | 98.5 |
|  | Piglet-C4 | OTU30 | 3 | *Methanobrevibacter millerae* | 98.3 |
|  | Piglet-C5 | OTU30 | 5 | *Methanobrevibacter millerae* | 98.2 |
|  | Piglet-C6 | OTU30 | 1 | *Methanobrevibacter millerae* | 98.3 |
|  | Piglet-C7 | OTU1 | 28 | *Methanobrevibacter smithii* | 99.1 |
|  | Piglet-C8 | OTU30 | 2 | *Methanobrevibacter millerae* | 98.5 |
|  | Piglet-C9 | OTU30 | 6 | *Methanobrevibacter millerae* | 98.4 |
|  | Piglet-C10 | OTU30 | 1 | *Methanobrevibacter millerae* | 98.0 |
|  | Piglet-C11 | OTU30 | 5 | *Methanobrevibacter millerae* | 98.1 |
|  | Piglet-C12 | OTU30 | 2 | *Methanobrevibacter millerae* | 98.1 |
|  | Piglet-C13 | OTU30 | 3 | *Methanobrevibacter millerae* | 98.3 |
|  | Piglet-C14 | OTU25 | 4 | *Methanobrevibacter millerae* | 98.2 |
|  | Piglet-C15 | OTU30 | 1 | *Methanobrevibacter millerae* | 98.1 |
|  | Piglet-C16 | OTU25 | 8 | *Methanobrevibacter millerae* | 98.1 |
|  | Piglet-C17 | OTU30 | 11 | *Methanobrevibacter millerae* | 98.3 |
|  | Piglet-C18 | OTU30 | 1 | *Methanobrevibacter millerae* | 97.8 |
|  | Piglet-C19 | OTU30 | 2 | *Methanobrevibacter millerae* | 97.9 |
|  | Piglet-C20 | OTU30 | 9 | *Methanobrevibacter millerae* | 98.2 |
|  | Piglet-C21 | OTU30 | 7 | *Methanobrevibacter millerae* | 98.2 |
|  | Piglet-C22 | OTU25 | 18 | *Methanobrevibacter gottschalkii* | 98.4 |
|  | Piglet-C23 | OTU30 | 1 | *Methanobrevibacter millerae* | 98.1 |
|  | Piglet-C24 | OTU30 | 3 | *Methanobrevibacter millerae* | 97.8 |
|  | Piglet-C25 | OTU30 | 1 | *Methanobrevibacter millerae* | 98.3 |
|  | Piglet-C26 | OTU4 | 3 | *Methanobrevibacter millerae* | 98.3 |
|  | Piglet-C27 | OTU2 | 4 | *Methanobrevibacter millerae* | 98.7 |
|  | Piglet-C28 | OTU10 | 6 | *Methanobrevibacter millerae* | 98.3 |
|  | Piglet-C29 | OTU30 | 1 | *Methanobrevibacter millerae* | 98.2 |
|  | Piglet-C30 | OTU30 | 1 | *Methanobrevibacter millerae* | 98.0 |
|  | Piglet-C31 | OTU30 | 1 | *Methanobrevibacter millerae* | 98.3 |
|  | Piglet-C32 | OTU31 | 1 | *Methanobrevibacter olleyae* | 95.9 |
|  | Piglet-C33 | OTU30 | 2 | *Methanobrevibacter millerae* | 98.2 |
|  | Piglet-C34 | OTU26 | 1 | *Methanobrevibacter millerae* | 97.2 |
|  | Piglet-C35 | OTU3 | 3 | *Methanobrevibacter millerae* | 98.1 |
|  | Piglet-C36 | OTU30 | 1 | *Methanobrevibacter millerae* | 98.3 |
|  | Piglet-C37 | OTU30 | 1 | *Methanobrevibacter millerae* | 98.3 |
|  | Piglet-C38 | OTU30 | 1 | *Methanobrevibacter millerae* | 98.3 |
|  | Piglet-C39 | OTU26 | 3 | *Methanobrevibacter millerae* | 97.5 |
|  | Piglet-C40 | OTU30 | 22 | *Methanobrevibacter millerae* | 98.6 |
|  | Piglet-C41 | OTU30 | 1 | *Methanobrevibacter millerae* | 98.1 |
|  | Piglet-C42 | OTU30 | 1 | *Methanobrevibacter millerae* | 98.2 |
|  | Piglet-C43 | OTU4 | 4 | *Methanobrevibacter millerae* | 98.6 |
|  | Piglet-C44 | OTU5 | 8 | *Methanobrevibacter boviskoreani* | 99.4 |
| Piglet-P | Piglet-P1 | OTU30 | 5 | *Methanobrevibacter millerae* | 98.3 |
|  | Piglet-P2 | OTU30 | 1 | *Methanobrevibacter millerae* | 98.3 |
|  | Piglet-P3 | OTU30 | 2 | *Methanobrevibacter millerae* | 98.3 |
|  | Piglet-P4 | OTU26 | 1 | *Methanobrevibacter millerae* | 97.1 |
|  | Piglet-P5 | OTU30 | 1 | *Methanobrevibacter millerae* | 98.2 |
|  | Piglet-P6 | OTU28 | 10 | *Methanobrevibacter ruminantium* | 98.8 |
|  | Piglet-P7 | OTU17 | 19 | *Methanomassiliicoccus luminyensis* | 88.0 |
|  | Piglet-P8 | OTU30 | 1 | *Methanobrevibacter millerae* | 97.9 |
|  | Piglet-P9 | OTU30 | 2 | *Methanobrevibacter millerae* | 98.4 |
|  | Piglet-P10 | OTU30 | 1 | *Methanobrevibacter millerae* | 98.3 |
|  | Piglet-P11 | OTU6 | 1 | *Methanobrevibacter millerae* | 97.5 |
|  | Piglet-P12 | OTU30 | 3 | *Methanobrevibacter millerae* | 98.2 |
|  | Piglet-P13 | OTU28 | 8 | *Methanobrevibacter ruminantium* | 98.9 |
|  | Piglet-P14 | OTU30 | 4 | *Methanobrevibacter gottschalkii* | 97.9 |
|  | Piglet-P15 | OTU30 | 2 | *Methanobrevibacter millerae* | 98.2 |
|  | Piglet-P16 | OTU30 | 1 | *Methanobrevibacter millerae* | 97.8 |
|  | Piglet-P17 | OTU30 | 8 | *Methanobrevibacter millerae* | 98.3 |
|  | Piglet-P18 | OTU30 | 1 | *Methanobrevibacter millerae* | 97.7 |
|  | Piglet-P19 | OTU30 | 2 | *Methanobrevibacter millerae* | 97.8 |
|  | Piglet-P20 | OTU30 | 3 | *Methanobrevibacter millerae* | 98.3 |
|  | Piglet-P21 | OTU30 | 1 | *Methanobrevibacter millerae* | 98.3 |
|  | Piglet-P22 | OTU30 | 4 | *Methanobrevibacter millerae* | 98.3 |
|  | Piglet-P23 | OTU30 | 6 | *Methanobrevibacter millerae* | 98.4 |
|  | Piglet-P24 | OTU7 | 27 | *Methanomassiliicoccus luminyensis* | 86.6 |
|  | Piglet-P25 | OTU26 | 4 | *Methanobrevibacter millerae* | 97.2 |
|  | Piglet-P26 | OTU30 | 2 | *Methanobrevibacter millerae* | 97.8 |
|  | Piglet-P27 | OTU8 | 1 | *Methanobrevibacter ruminantium* | 96.9 |
|  | Piglet-P28 | OTU30 | 3 | *Methanobrevibacter gottschalkii* | 98.3 |
|  | Piglet-P29 | OTU17 | 4 | *Methanomassiliicoccus luminyensis* | 88.1 |
|  | Piglet-P30 | OTU30 | 9 | *Methanobrevibacter millerae* | 98.6 |
|  | Piglet-P31 | OTU9 | 2 | *Methanobrevibacter boviskoreani* | 99.7 |
|  | Piglet-P32 | OTU30 | 1 | *Methanobrevibacter gottschalkii* | 97.9 |
|  | Piglet-P33 | OTU20 | 3 | *Methanomassiliicoccus luminyensis* | 87.1 |
|  | Piglet-P34 | OTU10 | 5 | *Methanobrevibacter millerae* | 98.3 |
|  | Piglet-P35 | OTU30 | 8 | *Methanobrevibacter millerae* | 97.9 |
|  | Piglet-P36 | OTU30 | 7 | *Methanobrevibacter millerae* | 98.2 |
|  | Piglet-P37 | OTU11 | 16 | *Methanomassiliicoccus luminyensis* | 84.6 |
|  | Piglet-P38 | OTU30 | 2 | *Methanobrevibacter millerae* | 98.5 |
|  | Piglet-P39 | OTU30 | 5 | *Methanobrevibacter millerae* | 98.1 |
|  | Piglet-P40 | OTU30 | 4 | *Methanobrevibacter millerae* | 98.3 |
| Finisher-C | Finisher-C1 | OTU17 | 3 | *Methanomassiliicoccus luminyensis* | 88.2 |
|  | Finisher-C2 | OTU30 | 4 | *Methanobrevibacter millerae* | 98.4 |
|  | Finisher-C3 | OTU30 | 1 | *Methanobrevibacter millerae* | 98.3 |
|  | Finisher-C4 | OTU30 | 2 | *Methanobrevibacter millerae* | 97.6 |
|  | Finisher-C5 | OTU25 | 2 | *Methanobrevibacter millerae* | 98.2 |
|  | Finisher-C6 | OTU14 | 1 | *Methanobrevibacter millerae* | 96.3 |
|  | Finisher-C7 | OTU31 | 9 | *Methanobrevibacter olleyae strain* | 96.1 |
|  | Finisher-C8 | OTU28 | 12 | *Methanobrevibacter ruminantium* | 98.7 |
|  | Finisher-C9 | OTU30 | 3 | *Methanobrevibacter millerae* | 98.3 |
|  | Finisher-C10 | OTU12 | 4 | *Methanobrevibacter ruminantium* | 96.4 |
|  | Finisher-C11 | OTU30 | 2 | *Methanobrevibacter millerae* | 98.0 |
|  | Finisher-C12 | OTU30 | 8 | *Methanobrevibacter millerae* | 98.3 |
|  | Finisher-C13 | OTU30 | 9 | *Methanobrevibacter millerae* | 98.3 |
|  | Finisher-C14 | OTU30 | 5 | *Methanobrevibacter millerae* | 98.3 |
|  | Finisher-C15 | OTU28 | 7 | *Methanobrevibacter ruminantium* | 98.6 |
|  | Finisher-C16 | OTU30 | 1 | *Methanobrevibacter millerae* | 98.1 |
|  | Finisher-C17 | OTU30 | 1 | *Methanobrevibacter millerae* | 98.3 |
|  | Finisher-C18 | OTU30 | 7 | *Methanobrevibacter millerae* | 98.2 |
|  | Finisher-C19 | OTU30 | 1 | *Methanobrevibacter millerae* | 98.2 |
|  | Finisher-C20 | OTU31 | 15 | *Methanobrevibacter smithii* | 96.1 |
|  | Finisher-C21 | OTU30 | 1 | *Methanobrevibacter millerae* | 98.3 |
|  | Finisher-C22 | OTU13 | 3 | *Methanobrevibacter ruminantium* | 97.7 |
|  | Finisher-C23 | OTU30 | 4 | *Methanobrevibacter millerae* | 98.2 |
|  | Finisher-C24 | OTU28 | 2 | *Methanobrevibacter ruminantium* | 98.6 |
|  | Finisher-C25 | OTU14 | 17 | *Methanobrevibacter smithii* | 96.7 |
|  | Finisher-C26 | OTU30 | 2 | *Methanobrevibacter millerae* | 98.0 |
|  | Finisher-C27 | OTU15 | 15 | *Methanomassiliicoccus luminyensis* | 88.0 |
|  | Finisher-C28 | OTU30 | 6 | *Methanobrevibacter millerae* | 98.3 |
|  | Finisher-C29 | OTU16 | 1 | *Methanobrevibacter millerae* | 95.5 |
|  | Finisher-C30 | OTU30 | 4 | *Methanobrevibacter millerae* | 98.3 |
|  | Finisher-C31 | OTU30 | 1 | *Methanobrevibacter millerae* | 98.1 |
|  | Finisher-C32 | OTU30 | 1 | *Methanobrevibacter millerae* | 98.2 |
|  | Finisher-C33 | OTU28 | 4 | *Methanobrevibacter ruminantium* | 98.7 |
|  | Finisher-C34 | OTU30 | 14 | *Methanobrevibacter millerae* | 98.1 |
|  | Finisher-C35 | OTU26 | 3 | *Methanobrevibacter millerae* | 97.0 |
|  | Finisher-C36 | OTU31 | 5 | *Methanobrevibacter smithii* | 96.0 |
|  | Finisher-C37 | OTU30 | 3 | *Methanobrevibacter millerae* | 98.0 |
|  | Finisher-C38 | OTU28 | 2 | *Methanobrevibacter ruminantium* | 98.7 |
|  | Finisher-C39 | OTU30 | 3 | *Methanobrevibacter millerae* | 98.3 |
|  | Finisher-C40 | OTU28 | 5 | *Methanobrevibacter ruminantium* | 98.8 |
|  | Finisher-C41 | OTU30 | 1 | *Methanobrevibacter millerae* | 98.3 |
| Finisher-P | Finisher-P1 | OTU17 | 7 | *Methanomassiliicoccus luminyensis* | 88.2 |
|  | Finisher-P2 | OTU30 | 1 | *Methanobrevibacter millerae* | 98.3 |
|  | Finisher-P3 | OTU30 | 3 | *Methanobrevibacter millerae* | 98.1 |
|  | Finisher-P4 | OTU30 | 1 | *Methanobrevibacter millerae* | 98.3 |
|  | Finisher-P5 | OTU30 | 1 | *Methanobrevibacter millerae* | 98.3 |
|  | Finisher-P6 | OTU26 | 5 | *Methanobrevibacter millerae* | 97.4 |
|  | Finisher-P7 | OTU30 | 2 | *Methanobrevibacter millerae* | 98.2 |
|  | Finisher-P8 | OTU31 | 1 | *Methanobrevibacter millerae* | 96.1 |
|  | Finisher-P9 | OTU18 | 2 | *Methanomassiliicoccus luminyensis* | 87.3 |
|  | Finisher-P10 | OTU30 | 38 | *Methanobrevibacter smithii* | 98.6 |
|  | Finisher-P11 | OTU19 | 8 | *Methanomassiliicoccus luminyensis* | 84.3 |
|  | Finisher-P12 | OTU20 | 4 | *Methanomassiliicoccus luminyensis* | 87.4 |
|  | Finisher-P13 | OTU21 | 2 | *Methanobrevibacter millerae* | 96.1 |
|  | Finisher-P14 | OTU30 | 1 | *Methanobrevibacter millerae* | 98.1 |
|  | Finisher-P15 | OTU30 | 3 | *Methanobrevibacter millerae* | 98.3 |
|  | Finisher-P16 | OTU30 | 7 | *Methanobrevibacter millerae* | 98.3 |
|  | Finisher-P17 | OTU27 | 4 | *Methanobrevibacter gottschalkii* | 97.6 |
|  | Finisher-P18 | OTU22 | 5 | *Methanobrevibacter gottschalkii* | 97.9 |
|  | Finisher-P19 | OTU23 | 4 | *Methanobrevibacter gottschalkii* | 97.5 |
|  | Finisher-P20 | OTU24 | 1 | *Methanobrevibacter gottschalkii* | 97.6 |
|  | Finisher-P21 | OTU30 | 1 | *Methanobrevibacter millerae* | 98.2 |
|  | Finisher-P22 | OTU30 | 1 | *Methanobrevibacter millerae* | 98.2 |
|  | Finisher-P23 | OTU30 | 1 | *Methanobrevibacter gottschalkii* | 97.9 |
|  | Finisher-P24 | OTU30 | 2 | *Methanobrevibacter millerae* | 97.9 |
|  | Finisher-P25 | OTU30 | 1 | *Methanobrevibacter millerae* | 98.2 |
|  | Finisher-P26 | OTU26 | 3 | *Methanobrevibacter millerae* | 97.8 |
|  | Finisher-P27 | OTU25 | 1 | *Methanobrevibacter millerae* | 98.4 |
|  | Finisher-P28 | OTU30 | 1 | *Methanobrevibacter millerae* | 98.3 |
|  | Finisher-P29 | OTU27 | 6 | *Methanobrevibacter gottschalkii* | 98.3 |
|  | Finisher-P30 | OTU30 | 1 | *Methanobrevibacter millerae* | 98.1 |
|  | Finisher-P31 | OTU30 | 3 | *Methanobrevibacter millerae* | 98.3 |
|  | Finisher-P32 | OTU26 | 2 | *Methanobrevibacter millerae* | 97.5 |
|  | Finisher-P33 | OTU30 | 1 | *Methanobrevibacter millerae* | 98.4 |
|  | Finisher-P34 | OTU26 | 1 | *Methanobrevibacter millerae* | 97.1 |
|  | Finisher-P35 | OTU27 | 2 | *Methanobrevibacter gottschalkii* | 98.3 |
|  | Finisher-P36 | OTU27 | 2 | *Methanobrevibacter gottschalkii* | 98.3 |
|  | Finisher-P37 | OTU28 | 7 | *Methanobrevibacter ruminantium* | 98.7 |
|  | Finisher-P38 | OTU29 | 19 | *Methanomassiliicoccus luminyensis* | 86.7 |
|  | Finisher-P39 | OTU30 | 4 | *Methanobrevibacter millerae* | 98.3 |
|  | Finisher-P40 | OTU30 | 6 | *Methanobrevibacter millerae* | 98.3 |
|  | Finisher-P41 | OTU30 | 1 | *Methanobrevibacter millerae* | 98.3 |
|  | Finisher-P42 | OTU31 | 21 | *Methanobrevibacter olleyae* | 96.1 |
|  | Finisher-P43 | OTU32 | 9 | *Methanobrevibacter gottschalkii* | 98.3 |

*Nearest valid taxon with the same name means the same strain.

**Table S2**. The alteration of methanogenic species, clades and functional gene copies of the three hydrogenotrophs in the colon of pigs (a summary)

| **Item** | | **Treat (PF vs Control)** | | **Time (Finisher vs Piglet)** | |
| --- | --- | --- | --- | --- | --- |
|  |  | **Piglet** | **Finisher** | **Control** | **Pea Fiber** |
| **Methanogen species** | *Methanobrevibacter millerae* | -22.53 | -18.34 | -23.86 | -19.85 |
|  | *Methanomassiliicoccus luminyensis* | **+36.32** | +11.13 | **+9.28** | -15.91 |
|  | *Methanobrevibacter smithii* | **-14.36** | +0.32 | +4.71 | **+19.39** |
|  | *Methanobrevibacter gottschalkii* | -5.02 | **+17.35** | **-9.23** | +13.14 |
|  | *Methanobrevibacter olleyae* | **-0.51** | +6.07 | +4.13 | **+10.71** |
|  | *Methanobrevibacter ruminantium* | +8.97 | -16.53 | +19.07 | -6.43 |
|  | *Methanobrevibacter boviskoreani* | -3.05 | 0.00 | **-4.10** | **-1.05** |
| **Methanogen clades** | SGMT | -41.73 | -0.67 | -28.38 | +12.68 |
|  | RO | +8.46 | -10.45 | +23.20 | +4.29 |
|  | SGMT+RO | -33.27 | -11.13 | -5.18 | -16.96 |
| **Functional gene copies** | *mcrA* | - | **↑** | - | **↑** |
|  | *dsrA* | **↑** | **↑** | - | **↑** |
|  | *fhs* | - | - | **↑** | - |

The alterations of methanogenic species and clades represented as %. “-” means decreased and “+” means increased. The background color of each cell indicates relative abundance of each phylum with red and green indicating increase and decrease. The alterations of the functional gene copies represented as arrows. “**↑**” means increased and “-” means no significantly change was detected.


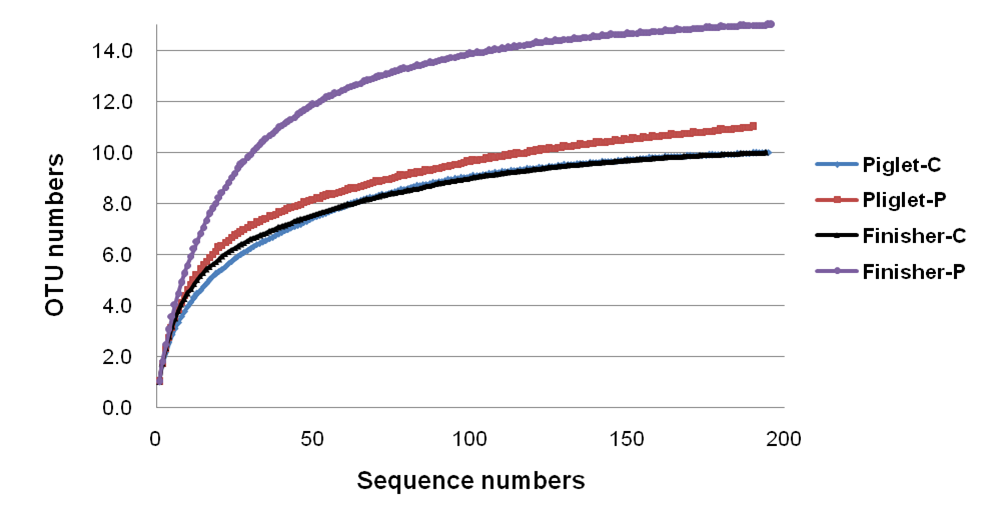


**Figure S1**. The rarefaction curves for the four clone libraries.


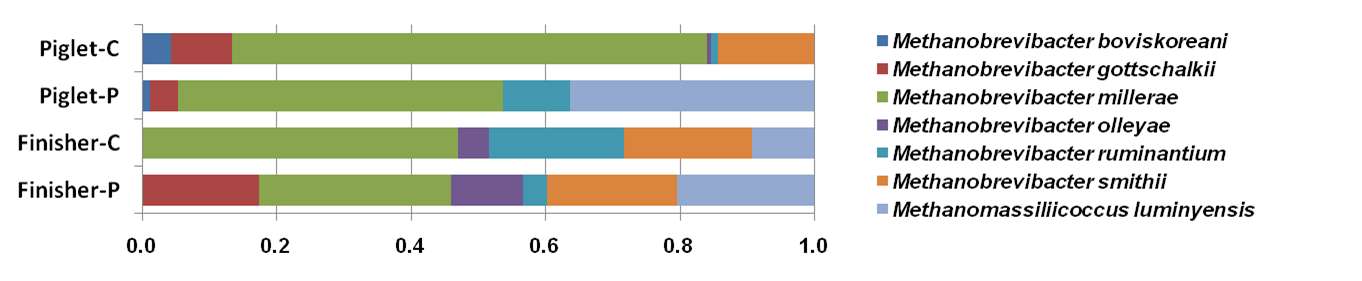


**Figure S2**. Stacked bar chart of the most similar methanogen species found in the four clone libraries. The values represent as % of total sequences.


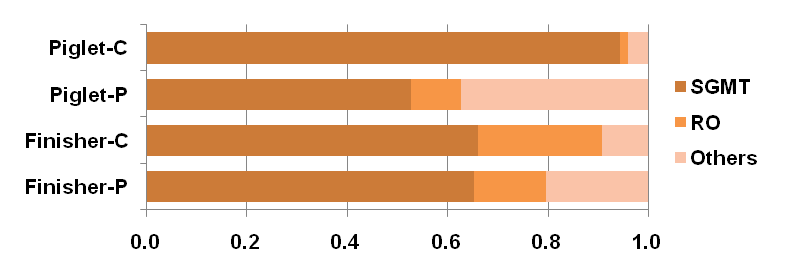


**Figure S3**. Stacked bar chart of the distribution of the two main methanogenic clades. The values represent as % of total sequences.
